# Current Trends on Protein Driven Bioinks for 3D Printing

**DOI:** 10.3390/pharmaceutics13091444

**Published:** 2021-09-10

**Authors:** Anabela Veiga, Inês V. Silva, Marta M. Duarte, Ana L. Oliveira

**Affiliations:** 1 CBQF—Centro de Biotecnologia e Química Fina—Laboratório Associado, Escola Superior de Biotecnologia, Universidade Católica Portuguesa, 4169-005 Porto, Portugal; anabelaveiga@fe.up.pt (A.V.); ines_3094@hotmail.com (I.V.S.); martasmduarte@gmail.com (M.M.D.); 2 LEPABE—Laboratory for Process Engineering, Environment, Biotechnology and Energy, Faculty of Engineering, University of Porto, 4099-002 Porto, Portugal

**Keywords:** bioink, extrusion biorpinting, protein-based

## Abstract

In the last decade, three-dimensional (3D) extrusion bioprinting has been on the top trend for innovative technologies in the field of biomedical engineering. In particular, protein-based bioinks such as collagen, gelatin, silk fibroin, elastic, fibrin and protein complexes based on decellularized extracellular matrix (dECM) are receiving increasing attention. This current interest is the result of protein’s tunable properties, biocompatibility, environmentally friendly nature and possibility to provide cells with the adequate cues, mimicking the extracellular matrix’s function. In this review we describe the most relevant stages of the development of a protein-driven bioink. The most popular formulations, molecular weights and extraction methods are covered. The different crosslinking methods used in protein bioinks, the formulation with other polymeric systems or molecules of interest as well as the bioprinting settings are herein highlighted. The cell embedding procedures, the in vitro, in vivo, in situ studies and final applications are also discussed. Finally, we approach the development and optimization of bioinks from a sequential perspective, discussing the relevance of each parameter during the pre-processing, processing, and post-processing stages of technological development. Through this approach the present review expects to provide, in a sequential manner, helpful methodological guidelines for the development of novel bioinks.

## 1. Introduction

Biofabrication has been defined as a process through which biomaterials with living cells and biological cues are used as building blocks to manufacture functional biological systems [1,2,3,4]. This field of cutting-edge technologies include three-dimensional (3D) bioprinting [5,6,7], electrospinning [8,9], or bio-plotting [10], which generate different functional constructs depending on the intended bio-medical application [11]. 3D bioprinting in particular, is one of the cutting-edge technologies available in this field which, according to the recent trends, has exponentially grown at the start of the year 2000 [4]. This technique enables the development of complex structures, such as organ-on-chip [12], scaffolds [13,14], and micro-tissues [15,16], through the deposition of materials according to a scripted design, with minute control over multi-material addition and distribution, patterning, printing speed, among others [2,3,4].

Three essential stages can be identified in most of the research articles in the literature that focus on bioprinting: a pre-processing stage, a processing or printing stage, and finally a post-processing stage. It is clear that it is not possible to have widely accepted processing methodologies as the materials, evaluated parameters and processing strategies are on a demand basis, considering the application [3,17,18].

To have a printable material that can cope with the processing challenges while holding the adequate environment for cell embedding is the most critical requirement. The concept of bioink has therefore been established, as a material that can comprise cells to be used in biofabrication methodologies such a 3D-printing [19]. The cell carrier material could also contain a myriad of components such as bioactive molecules. This concept establishes cells as a mandatory component of the material which can be distinguished from other inks that use cell seeding after fabrication [19]. The bioink will be comprised of encapsulated cells and carrier material with or without added components and crosslinking agents. Moreover, it should provide essential biochemical and mechanical cues that are known to regulate and modulate cell fate [20]. Bioinks should then be able to (1) provide a chemically similar micro-environment to the native/targeted tissue while (2) being biocompatible and have (3) a controlled biodegradability to allow for cellular remodeling. Since cellular encapsulation is a required feature, the bioink should also present (4) shear-thinning behavior that allows extrusion with minimal stress to the encapsulated cells, (5) sol-gel transition, and (6) extrudability, the ability to be extruded without clogging the nozzle [21,22]. Additionally, the resulting printed structure should provide similar (7) mechanical strength and rigidity, adequate (8) shape fidelity with proper stacking ability to provide a 3D environment, (9) material density and pore size for optimal diffusion of oxygen, carbon dioxide and nutrients [21,22]. Crosslinking type can play a crucial role in modulating properties (7, 8, and 9) but it can also hinder cell capacity. As such, it should be carefully considered (e.g., long exposure to UV irradiation, a typical photocrosslinking technique that frequently has been associated with DNA damage, hypoxia conditions and can be deleterious to materials properties) [21,22,23,24].

Bioinks, like other biomaterials, can be divided into natural or synthetic. Synthetic materials typically used in 3D printing are polycaprolactone (PCL), polyethylene glycol (PEG), Pluronic, and Polyvinylpyrrolidone (PVP) [21]. These materials have some advantages such as their ease of handle, high control over their chemical and physical properties, or batch-to-batch reproducibility. However, synthetic-driven materials are bioinert, often have poor biocompatibility [21], as they usually lack cell binding or recognition sites, and have absence of topographical and biochemical cues to promote cell proliferation and differentiation [25]. 

On the other hand, natural-based bioinks comprise a larger array of current explored options, namely polymeric-based materials [21,26,27]. Among these, protein-based materials such as collagen [28,29], fibrin [30,31,32], keratin [33,34], or decellularized extracellular matrix-based materials [35,36], have been broadly used in several biomedical fields and more recently integrated in 3D bioprinting systems due to its abundance, low cost, tunable physicochemical, mechanical and biological properties and excellent biocompatibility and biodegradability [25,27,37] While these materials are generally more difficult to manipulate, often possessing poor mechanical properties, or unpredictable behavior when printed, they can provide a better environment for cell growth as a result of specific encoded designs, i.e., amino acid sequence information which guides the construct assembly and mimic the extracellular matrix (ECM) [17,18,21,37]. Protein-based materials can additionally be used to modify several rheological and biochemical properties of bioinks, allowing for higher construct fidelity [38,39,40]. A final consideration, of special importance in modern science, is that these materials are both renewable, and very environmentally-friendly, particularly when compared to fossil-derived synthetic polymers [40]. Nonetheless, they can be highly sensitive (frequently thermolabile) and therefore difficult to extract and purify.

**Figure 1:**
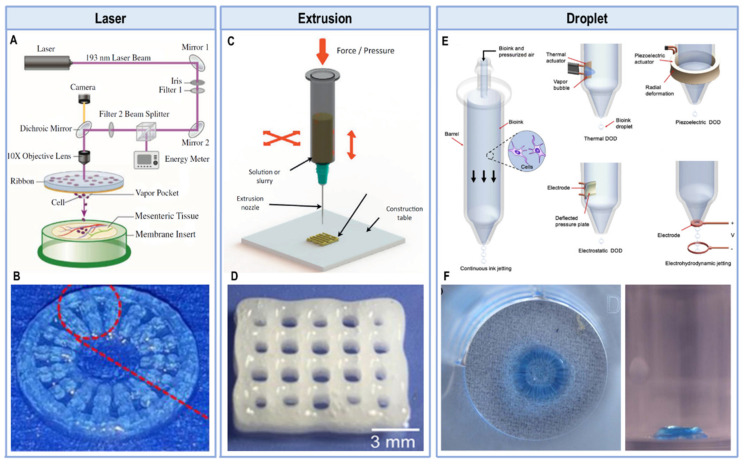
3D bioprinting technologies and printed constructs (adapted with permission from [11,15,43,44,45,46]).

The most commonly used bioprinting techniques can be divided in extrusion, laser-based, and droplet ejection [11,18,21] (Figure 1). Of these available methodologies, extrusion-based bioprinting is one of the leading manufacturing techniques in tissue engineering applications [41]. This method consists on automated dispensing a bioink either through mechanical force done by a screw or piston, or pneumatically by using gas or pressurized air. The bioink is then extruded continuously in the form of a strand [42]. Extrusion-based bioprinters can print with higher cell densities when compared to other methods, and usually allow for the use of multiple printheads and materials withing a single construct [37,41,42]. This allows for the construction of very complex materials, as researchers can manipulate regional differences in cell density, types of materials or cells used, and even use signaling molecules [41,42]. However, this technique still comes with some drawbacks like the lack of available bioinks, and the high shear stress cells can get exposed to, which can reduce cell viability in the final construct [41]. Thus, experimenting with different crosslinking methods, material formulation and additional components to improve the overall mechanical, rheological, physicochemical and biological properties is crucial in these systems [3,40].

In the present review, the most recent trends in protein-based bioinks are covered, highlighting protein origin, extraction methods and resulting properties of this class of bioconstructs. In addition to the conventional preprocessing parameters, processing techniques such as crosslinking strategies, the addition of bioactive molecules, the relevance of rheological properties, printing settings, and cell embedding requirements and limitations are discussed. Based on these findings, a logical sequence for the development and optimization of bioinks is proposed that can be used as a useful platform for the development and optimization of innovative bioinks.

## 2. Protein-Based Bioinks

### 2.1. Pre-Processing Methods

#### 2.1.1. Bioink Formulation

**Figure 2:**
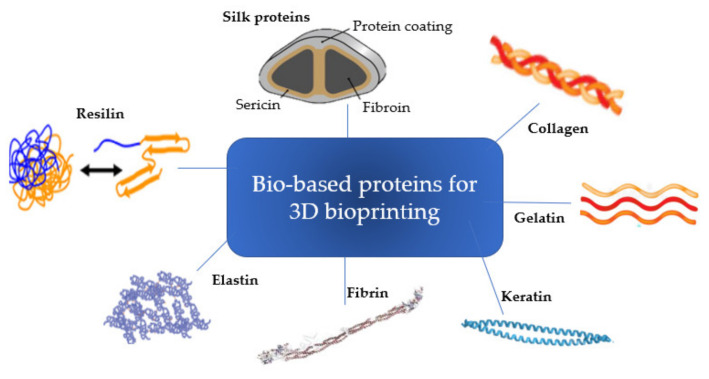
Most used proteins for biomedical engineering.

The core material and concentration in a bioink formulation are key parameters that must be considered in the pre-processing phase to assure reproducibility of the process, and to increase printability. The protein screening, in addition to varying according to the final application, depends on the origin, structure, composition and characteristics of the designated material. Natural structural proteins display critical physiological and biological cues for the development of bioinks. Besides being environmentally friendly and renewable, protein-based materials tend to have also excellent biocompatibility, strength, elongation, toughness, slow degradability [26,27] (Table 1) (Figure 2).

Fibrous proteins such as elastin, collagen, keratin, silk fibroin, fibrin and resilin are characterized by highly repetitive amino acid sequences that provide mechanical and architectural functions in nature [47]. Although these are amongst the most used proteins for biomaterials, other nonstructural proteins, such as silk sericin, are gaining momentum over the last few years [26,48]. In this particular globular protein, the hydrophilic amino acid side chains lie on the surface exposed to the water, making the protein soluble in aqueous solutions and suitable for cellular signaling, binding, regulation, transport and catalysis [27]. For bioprinting, these proteins are used to develop 3D hydrogels due to its good printability and improved better medical/clinical behavior when bioprinted [3]. Even though most of the covered proteins have already been used in numerous works to obtain hydrogels for bioengineering studies, not all of them have been used as a bioink for 3D extrusion bioprinting systems [33,34,49,50,51].

Collagen is the principal structural protein present in connective tissues, constituting 25% to 35% of protein in the human body. Twenty-nine types of collagens composed of at least 46 distinct polypeptide chains have been identified so far and classified primarily according to their structure. Over 90% of the collagen in the body is type I, while the other common types of collagen include types II, III, and IV [52]. Thus, the majority of collagen hydrogels are produced from type I, that under physiological conditions (neutral pH and 37 °C) starts to self-organize into fibrils that present good tensile strength and flexibility and can be further assembled and cross-linked so as to support mechanical stimulation [28]. However, collagen-based bioinks have low collagen concentrations (rarely exceeding 5% *w*/*v*) and as a result they present low mechanical properties [29].

If collagen is irreversibly hydrolyzed, it is known as gelatin. Gelatin has a low molecular weight and presents a less organized arrangement. Nevertheless it shares a very close molecular composition to collagen type I [53]. Thanks to its versatile physicochemical properties, gelatin allows the development of both high and low viscosity gelatin based bioinks. The gelling can be controlled by temperature, because the hydrogen bonds that hold the triple-helix conformation of gelatin together are weakened by increased temperature, generally above 30 °C, which facilitates optimization of the flow behavior during bioprinting [40,54]. Gelatin concentrations in a bioink can range from 1–20%, being 5% *w*/*v* the most common value [3].

Keratin is the name for a family of structural proteins which are abundant in vertebrate epithelia and corneous tissues. It consists of many parallel polypeptide chains with alpha- helix or beta-helix conformations [55]. ‘Soft’ keratins composition includes bundles of fibers that self-assemble as a result of crosslinking through sulfur-sulfur bonds involving the cysteine side chains, forming porous gels [56]. Most published works use keratin for the development of scaffolds or hydrogels for 3D printing but not as a bioink main component [33]. This protein has been used in few works in a lithography-based 3D printer and in digital light processing 3D printing [33,34]. Although to our knowledge, extrusion systems with keratin are not yet reported, there are already several works in which well-defined structures are obtained by self-assembling casting [57] and photo-crosslinking [58] by adding a photosensitive compound (such as riboflavin-SPS sodium persulfate-hydroquinone) in the solution and then using UV to induce the formation of a link between the monomers [33]. Moreover, in the work of Wang et al. [59] a keratin-based hydrogel polymerized with Ca^2+^ showed that cell viability is persevered and that cells remain clustered in proliferative colonies within the hydrogels, showing the potential of this protein to obtain a bioink that also acts as a substrate for cell culture in 3D extrusion bioprinting [59].

Fibrin is a key component of the coagulation cascade and is derived from soluble circulating fibrinogen, a glycoprotein synthetized in the liver made by three pairs of polypeptide chains, designated Aα, Bβ and γ. In the presence of the serine protease thrombin, released in response to vascular injury, fibrinogen is hydrolyzed and polymerizes into fibrin [18]. Fibrin contains numerous binding sites for cells and growth factors that promote platelet spreading, cell infiltration, fibroblast proliferation, and angiogenesis [60]. Despite its interesting properties, fibrin adopts a Newtonian fluid behavior, in which the viscosity of fibrinogen solution remains constant with shear variation, meaning that this protein is not capable of sustaining a stable 3D bioprinted shape, regardless of its concentration [61]. Thus, this protein is usually incorporated with other polymeric systems [30,31,32].

Elastin is most abundant in tissues where elasticity is of major importance. This unstructured extracellular matrix protein includes a wide range of elastic peptides and protein sequences that exist in different lengths and with different compositions [56]. The elasticity and resilience of elastin results from a combination of polymer chain recoil and a highly cross-linked structure [37]. This protein can be used in incorporation using different materials such as methacryloyl-substituted recombinant human tropoelastin (5% *w*/*v*) [62], elastin-like recombinant (2–5% *w*/*v*) [62,63].

Resilin is an elastomeric protein found in many insects and other arthropods. It is composed of randomly orientated coiled polypeptide chains that are covalently cross-linked together at regular intervals by the two unusual amino acids dityrosine and trityrosine forming a stable network with a high degree of flexibility and mobility [64]. This protein can be used as an additive to improve a biomaterial mechanical performance [65]. Despite the fact that resilin-based bionks haven’t yet been developed, cell encapsulation of human aortic adventitial fibroblasts in resilin-like polypeptides [49] and human bone marrow-derived mesenchymal stem cells (hMSCs) with cell viability for an extended time of at least 21 days [50], have been reported.

Silk has been used in the textile industry for centuries due to its aesthetic quality and thermal properties as a clothing material and more recently for biomedical applications. When considering the most used silk source, the cocoon based silk, there are two types of proteins that can be explored: fibroin a fibrous semicrystalline protein (70~75% crystallinity with corresponding 25~30% amorphous region) which is the core filament and the surrounding gluing agent, sericin (25–30% of the weight of the fiber) [26,55,66]. Silk fibroin has been used as a bioink in concentrations up to 30% *w*/*v* [67]. On the other hand, unmodified silk sericin does not self-assemble strongly enough to be used as a hydrogel. Therefore, interlink silk-sericin with peptide chains or the strengthen of their structural cohesion is essential for the production of hydrogels and further development into a bioink [51]. For example, an interesting strategy is to use enzymatic crosslinking as evidenced in the work of Baptista-Silva et al. [68], where a sericin hydrogel was obtained in approximately 3 min under physiological conditions, via peroxidase mediated cross-linking.

In addition to using isolated proteins as the main component, there are protein-based bioinks that are based on protein complexes deriving from decellularized extracellular matrix (dECM) [69]. The ECM has a unique composition that is generated during tissue development through the orchestrated participation of various cellular components. The dECM major elements are collagen, glycosaminoglycans (GAGs), fibronectin, (tropo)elastin and laminin [70]. The advantages of using dECM is that it is free from xenogeneic and allogeneic cellular material [71], while being is tissue-specific, therefore providing biochemical cues that regulate and modulate cell fate into the desired tissue type [70,72,73]. Recently, several bioinks have been proposed using dECM as the hydrogel main component or added in small amounts to the polymeric matrix to provide instructive cues for a specific cell fate [69]. Zhang and colleagues [74] mixed silk fibroin (SF) and cartilage dECM to achieve a matrix for cartilage regeneration that could provide mechanical strength and biological activity of the native tissue. Ideal bioink printability was found with a 2 ≤ dECM % *w*/*v* < 3 and silk fibroin between 2 ≤ SF % *w*/*v* < 7.5. However, Jorgensen et al., verified that 1% skin dECM combined with fibrinogen present better printability than 2% skin dECM [75]. Additionally, Kim and college verified better cell viability and stable structure formation for 10 mg/mL SIS dECM-collagen when compared with other concentrations of dECM [76]. Therefore, ideal dECM concentration should be optimized on a dECM tissue by tissue basis while also considering the concentration of added proteins.

**Table 1:** Main origin, main characteristics and applications of proteins used in the development of 3D materials and bioinks.

Protein	Origin	Extraction	Structure	Amino Acid Sequence	Main Characteristics	Main Applications	Main Challenges for 3D Bioprinting	Ref.
Collagen	Animal tissue, including tendons, ligaments and skin	- Neutral saline solutions, acid solutions, and acid solutions with added enzymes.	- trimeric molecules (polypeptide α-chains) - triple helix-tertiary structure	- (GPX)n, where X is any amino acid other than glycine, proline or hydroxyproline	- high tensile strength and flexibility - cell adhesive cues - low mechanical properties Gelatin: - improved biodegradability and biocompatibility - Temperature responsive	- Skin tissue engineering (bone, cartilage, skin, liver, nervous system models, cornea) - Food additives - Cosmetics	- low mechanical properties	[28,29]
Gelatin		Obtained by partially hydrolyzing collagen under the action of an enzyme such as neutrase or under the action of an acid or an alkaline.	- random coiled domains				- poor rheological properties	[3,40,54]
Keratin	- wool, horn, hair, hooves, shells, beaks, fingernails, toenails, feathers, and claws	- by oxidative and reductive chemistry	Twisted helices	- CCXPX and CCXS(T)S(T)	-high stability - low solubility	- Cosmetic (hair products) - Soft tissue regeneration	- low extensibility - insolubility	[33,34]
Fibrin	- blood	- isolation and concentration of blood fibrinogen by centrifugation combined with cryoprecipitation.		- (GPRP)_n_ and (GHRP)n	- elastic and viscous properties - Short gelation time	fibrin-based sealants	- suitable crosslinking required - poor mechanical properties and rapid degradation	[30,31,32,61]
Elastin	- lungs, blood vessels, aorta, and skin	Animal-derived tropoelastin, recombinant production	β-spirals	- (VAPGVG)_n_	- flexibility	- Skin tissue engineering (vascular grafts, heart valves and elastic cartilage)	- elastin purification is required. During this purification process, contaminations often take place.	[62,63,77]
Resilin	- insect cuticles		β-turns	-(AQTPSSQYGAP)_n_	- rubber-elasticity - high mechanical properties	- conductive, elastic and adhesive hydrogels suitable as biosensor	- difficult to identify the primary sequence and molecular structure of resilin due to the reduced stability during purification	[49,50,78]
Silk Fibroin	Domestic silkworms: *Bombyx mori*; wild silkworms: *Antheraea pernyi* and *Samia cynthia ricini*; Spiders: *Nephila clavipes* and *Araneus diadematus*; recombinant silk proteins in different host systems	- Dissolving in a concentrated solution of lithium bromide or in a ternary solvent system of calcium chloride/ethanol/water	β-sheet structures connected by amorphous links	- Silk fiber: (GGXaa)_n_ - Xaa = A, Y, L or Q - predominance of glycine and alanine in fibroin predominance of serine in sericin	- structural integrity,	- Skin tissue engineering - sutures	- low mechanical parameters, and high enzymatic degradation rate.	[26,55,66,67]
Silk Sericin		- reaction types (acidic, alkaline, enzymatic) and different conditions (time, temperature, pressure, pH)	amorphous random spiral/β-sheets		- antioxidant, antibacterial, UV-resistant, and ability to release moisturizing factors	- Cosmetic (creams) - Skin tissue engineering - Bone tissue engineering (regulate hydroxyapatite biomineralization process)	- poor structural integrity	[51,68]
Decellularized extracellular matrix (dECM)	allograft and xenograft tissue	Combination of decellularization methods (chemical, biological and physical) applied using different tecnhqiues (perfusion decellularization, pressuregradient, supercritical fluid, or immersion and agitation)	n/a	n/a	- Improves and regulate cellular functions of specific tissue duo to naturally present growth and differentiation factors - Decrease risk of immune-mediated rejection	- In vitro diseased tissue models - Tissue repair - Tissue replacement	- define the most suitable formulations	[79,80,81]

#### 2.1.2. Extraction Methods

An aspect that should be considered when developing a protein-based bioink is the extraction method used to obtain the necessary degree of purification while assuring the best quality possible. The major concern related to protein-based materials is that molar mass distribution, structure, composition, and the subsequent functional features heavily depend on the raw material and extraction technique used. The extraction and purification methodologies can degrade the protein which will critically affect the molecular weight (MW) of proteins, which is intrinsically connected to its rheological properties. Above a critical MW, viscosity is proportional to MW^3.4^ due to entanglement of the polymer molecules [82,83].

Porcine skin-derived type-I collagen is usually used in bioink formulation, with a MW of about 250–300 kDa [84,85]. Even at low concentrations, collagen has high viscosity due to the strong electrostatic repulsion among the molecular chains. On the other hand, hydrolyzed collagen is composed by a variety of peptides with low molecular weight (3–6 KDa) hence having low viscosity, independently of concentration [86]. Fibrinogen can be obtained from bovine plasma (65–85% protein, 75% clottable protein) with MW of 63.5 kDa and 56 kDa for α-chain and β-chain, respectively. Elastin is usually synthesized and purified by *Escherichia coli* recombinant expression system, resulting in a MW of 37–60.6 KDa [63,87,88]. The monomer unit contains a VPGIG sequence that confers the mechanical properties (similar to natural elastin) and the biocompatibility and the stimuli-responsive nature. VPGKG building block present in the structure is a modification of the first, containing lysine, so that the lysine ε amino groups can be used for crosslinking [89]. While these proteins are usually acquired by a supplier, that indicates the characteristics of the material, silk fibroin extraction and purification is usually performed as part of the pre-processing due to the metastable character of the protein solutions. Cocoons from the silkworm *Bombyx mori* have been used as the most standardized source (Table 1). In the literature, most of the fibroin used as a bioink has a MW which ranges from ~400 to 80 KDa, but gel point increase is associated with lower MWs [90]. Thus to ensure batch-to-batch stability, it is crucial to follow well-defined and standardized protocols in the pre-processing stage, that guarantee the reproducibility of results [40,55].

#### 2.1.3. Preliminary Evaluation Techniques

To guarantee that the formulations are suitable for 3D bioprinting, the material must be further characterized using simple quantitative measurements to assess printability, mechanical and in vitro behavior. The materials are selected for its biocompatible components and favorable rheological properties that allow extrusion, recovery, and maintenance of the shape of the bioprinted layers in order to achieve 3D architectural integrity (Table 2). These parameters depend not only of the core protein selected for the bionk development, but also on the addition of other components and crosslinking method used [3,40,91].

#### 2.1.4. Crosslinkers

During the process of 3D bioprinting, protein-based solutions are transformed into 3D constructs via crosslinking. This is a key procedure that significantly influences the mechanical and physicochemical characteristics of the bioprinted structures and the cellular behavior of embedded cells. This process is particular important in extrusion bioprinting, since this process can affect the rheological properties of the material [92].

Although there is still no agreement on the different crosslinking classes [3,92] usually crosslinking methods are divided into four types: thermal (controlled by temperatures changes), chemical (controlled by the addition of reacting agents that result on the formation of nonreversible covalent bonding between polymeric chains), physical (triggered by physical procedures, usually ionic interaction or UV light that leads to the formation of noncovalent bonds, such as hydrogen bonds, hydrophobic interactions, electrostatic attraction and ionic crosslinking), enzymatic (enzymes used as catalysts to promote the formation of covalent bonds) or a combination of the previous [3,92] .

Thermal methods are one of the simplest methods of crosslinking and should be conducted at 37 °C during the printing. This procedure implies setting a slightly higher temperature to obtain physiological temperature at the nozzle tip or performed using room temperature (20–25 °C). Protein-based bioinks incorporating polysaccharides such as alginate [93] and agarose [94] can be printed by this process [3]. In this particular cases the temperature-induced gelation is faster than the Ca^2+^ induced gelation, which can improve the initial stability of the printed construct [95,96]. However, the degree of crosslinking cannot be precisely controlled, and overheating can affect cell viability inside the bioink [92]. 

Physical crosslinking can be of ionic nature, for example, using Ca^2+^ cations (CaCl_2_, CaSO_4_, NaCl_2_, CaCO_3_). A common procedure, especially in protein/alginate systems, is to dip the 3D construct into an ionic bath after bioprinting to induce gelation [95,97,98]. For example gelatin/alginate-based bioinks often suffer double crosslinking using a CaCl_2_ (1–10% *w*/*v*) bath after the initial thermal polymerization to harden the final material [95]. However, excessive amounts of Ca^2+^ can hinder cell viability and thus the work of the group of Sun [99], a range of 1.5/0.5% *w*/*v* for polymer/CaCl_2_ was established as the most adequate in order to avoid toxicity, for their developed chitosan/alginate bioink formulation [100]. These findings were corroborated by Demitas et al. [100] where bioprinted hydrogels were immersed in CaCl_2_ for a maximum of 15 min to avoid subsequent toxic effects. In other work presented by Saarai and co-authors, to remove the excess of Ca^2+^, the developed constructs were submitted to a washing step [101].

Physical crosslinking can also include other non-covalent interaction. Recently, tannic acid has also been used as a natural crosslinker in collagen-based bioinks (1–6 wt%), through the formation of hydrogen bonds [102,103]. Crosslinking time of around 10 min was reported to be sufficient independently of the concentration used [102]. Kim and colleague observed that SIS dECM-collagen bioink crosslinked with <2 wt% tannic acid produced mechanically stable villus and high cell viability (~90%) [76].

Chemical crosslinking approaches can be achieved by adding a photoinitiator (e.g., Irgacure D-2959, LAD-lithium phenyl-2,4,6-trimethylbenzoylphosphinate, Eosin Y, ruthenium/sodium persulfate, Rose Bengal) to protein/GelMA bioink formulation [104]. Despite the resulting good elastic properties and ease of use, this crosslinking strategy, exposing the encapsulated cells to UV light may damage its DNA [105]. To eliminate this effect, Lee and co-authors [62] have used light-emitting diode (LED) to photo polymerize Gelatin methacryloyl (GelMA)/methacryloyl-substituted recombinant human tropoelastin bioprinted structures (405 nm). The resulting structures had a tensile modulus of 47.9 ± 2.6 kPa [62]. Mau et al. [15] followed a similar approach to developed a gelatin-dECM microtissue. The authors verified that adding liver dECM decreased photo-crosslinking curing time which led to better shape fidelity and provides higher viability of encapsulated cells associated with decrease UV light exposure time, from 3.5 s (gelatin control) to 2.5 s. Enzymes can be used as catalysts to promote the formation of covalent bonds between protein-driven bioinks. This method is very appealing in natural systems as the mildness of the enzymatic reactions are favorable for living cells. By adding tyrosinase to silk fibroin/gelatin bioinks, stable rheological properties were obtained after 1 h (1.2 and 1.3 Pa·s at 100 s^−1^) [97,106]. Thus, longer printing time windows may hamper cell survival during the fabrication process.

Horseradish peroxidase (HRP)/hydrogen peroxide (H_2_O_2_) crosslinking has also been used for protein-based bioinks as a mild strategy to create cell-friendly microenvironments. In Schwab et al. [107] research work, tyramine derivatives of hyaluronan cross-linking and fibrillogenesis occur simultaneously, thus avoiding phase separation [107]. Gelation time, stiffness and degradation rate, can be easily manipulated by varying the concentrations of HRP and H_2_O_2_ [108].

The proteins self-assembly mechanisms have also been explored as a natural form to obtain a crosslinked structure. In particular, fibrin/gelatin-based bioinks use enzymatic crosslinking of the fibrinogen by thrombin [32,109,110] As a thermoreversible biomaterial, the blending of gelatin with fibrinogen will increase its elasticity after cooling. As the interlinking of its fiber network is caused by hydrogen bonding, it can undergo a transition from a solidified gel to a liquefied solution by rising the temperature from low such as 4 °C to high such as 25 °C [109]. In this bioinks, genipin can be added to avoid crosslinking of the polymers contained in the cross-linker solution before printing [30,111].

In addition to the traditionally used crosslinking methods, there are other chemical and physical-based approaches. In 2021, a versatile biorthogonal bioink crosslinking mechanism was proposed by Hyll and co-workers [88]. Strain-promoted azide-alkyne cycloaddition reaction between azides and bicyclononynes was used to allow formation of covalent bond between two distinct, complementary chemical functional groups. This bioink was successfully used in recombinant elastin-like protein. Elastin bioinks can also be non-covalently crosslinked with graphene oxide (0.1 wt%) to obtain a liquid-in-liquid bioink as showed in the work conducted by Wu et al. [63]. Contrary to conventional extrusion systems, in liquid-liquid bioprinting, multicomponent interfacial diffusion-reaction self-assembly is achieved by the inoculation of one low viscosity liquid phase into another [112]. This method is often applied to obtain complex capillary-based constructs (resolutions down to ∼10 µm in diameter made with ∼2 µm walls [63]. This system can also be implemented in alginate-based materials such as gelatin/alginate bioinks to directly print the polymer into a cross-linking solution of calcium chloride [113].

#### 2.1.5. Conjugation with Other Molecules/Polymers

Conjugation with other biopolymeric systems is a common practice to produce materials with improved properties and achieve control of functional and biological features. Therefore, many approaches have been explored to combine the natural proteins or genetic engineered proteins with other materials to produce special characteristics and generate multifunctional and biodegradable composite biomaterials [26].

Among these, the addition of polysaccharides such as alginate, agarose, chitosan and hyaluronic acid to obtain improved rheological and biological properties can be adopted. Alginate is a naturally occurring anionic polymer composed of β-d-mannuronic acid and α-l-guluronic acid joined by a 1–4 glycosidic bond, typically obtained from brown seaweed (*Phaeophyceae*) [114]. The MW can range from 32–400 g/mol with increasing viscous properties [115]. As alginate is quickly, but reversibly, crosslinked by Ca^2+^, it has been employed as a sacrificial template and is among the best natural polymers in terms of rheologic [3,116]. In the work of Ratanavaraporn et al. [117] a silk fibroin/alginate bioink was developed with different alginate concentrations (0.5% to 1% *w*/*v*). An increase in sodium alginate concentration from 0.5% to 1% resulted in better uniformity and higher stiffness of the bioink filaments [117]. Higher concentrations of alginate in gelatin, collagen and fibrin-based bioinks have been reported (7 % *w*/*v*) [98,111,118]. However, as cell-binding motifs are not present in this polymer, lower concentrations are most commonly used [97,119].

Agarose is a polysaccharide consisting of alternating β-d-galactose and 3,6-anhydro-l-galactose units of agarobiose, generally extracted from certain red seaweed (MW 12 ≈ kDa). The thermo-reversible gelation behavior of this material as well as its adjustable water adsorption capacity that supports cell activity is used in 3D bioprinting technology [120]. By mixing agarose, collagen-based bioinks become more stable [121,122]. In the study of Köpf et al. [123], different agarose concentrations were evaluated (0.25–1.5% *w*/*v*), being 0.5% *w*/*v* associated with better cell spreading and 1.5% *w*/*v* with improved printing accuracy [123].

Chitosan is a linear polysaccharide with β-(1–4)-linked D-glucosamine and N- acetyl-d-glucosamine chemical structure that can be found in exoskeleton of invertebrates and fungi. Collagen with low MW chitosan (50–190 KDa) has been used as hydrogel for 3D extrusion bioprinting, demonstrating a shear-thinning behavior, with viscosity values at low shear rates between 0.35 and 2.80 Pa.s (0.5, 1, 1.5 % *w*/*v*) [124]. However, cell incorporation is still a challenge in these systems [37].

Hyaluronic acid is a linear anionic polysaccharide consisting of alternating units of N-acetyl-d-glucosamine and glucuronic acid making it a member of the glycosaminoglycan family. This biocompatible, biodegradable and bioresorbable material allows for easy diffusion of nutrients. Furthermore, hyaluronic acid with MW of 20–200 kDa takes part in biological processes such as wound healing [125]. Nevertheless, it lacks low stability and mechanical properties [126] and thus is used with other components such as fibrin and gelatin and further enzymatic and photo-crosslinking (3 g/L) [110]. Several collagen based studies have focused on the incorporation of dual crosslinkable hyaluronic acid such as tyramine-modified hyaluronic acid (280–290 kDa) to obtain collagen fibril orientation at the microscopic level [107,127,128,129]. Glycerol, an organic polyol compound can also be added to enhance the spatial resolution and uniformity of the printed patterns [130]. As large amount of this component can affect cell viability, low concentrations can be used (up to 10% *v*/*v*) [130].

The addition of nano-structured materials (of metallic, polymeric and ceramic nature) is also a strategy to enhance the overall characteristics of protein-driven bioinks [84,131] To accelerate the orientation of cells and mimic the electrical properties of muscle tissue, Kim et al. [131] presented a collagen bioink with gold nanowires that were aligned by applying shear stress via a microsize nozzle and an electric field after bioprinting. In order to reach a similar result, the same research team used tricalcium phosphate (TCP) to obtain fibrillated collagen, exhibiting enhanced osteogenic activities compared to the pure collagen bioinks [84]. Nano structures can also have other purposes such as substituting Pluronic, the conventional sacrificial material, as demonstrated by Clark an co-authors that proposed self-supporting gelatin nanoparticles capable of creating large hollow structures through bioprinting [129].

Growth factors such as TGF-β3 have also been added to bioink systems to improve its biological properties. Silk fibroin/dECM with added TGF-β3 significantly enhanced the deposition ratio of ECM, that surpassed the degradation ratio of the scaffold which in turn helped prevent the shrinkage of the construct [74].

Synthetic polymers can also be added to protein-based hydrogels to improve the overall mechanical properties of the resulting bioink. Gelatin is often used in the form of gelatin-methacryloyl (GelMA), which is created through a gelatin and methacrylic anhydride reaction (<7% *w*/*v*) [37]. The main advantage of GelMA is that it does not require crosslinking agents or localized gelation during extrusion printing, but it does require the use of a photoinitiator followed by UV light exposure, which can hamper decreased cell viability [37]. Several fibroin [104], elastin [62], collagen [128] and gelatin [132] bioinks with GelMA have been reported.

Other nanocomponents that can be added to protein-based bioinks include gellan gum, dextran, cellulose, Matrigel [37], polyethylene glycol (PEG) [3] and supporting and sacrificial materials like polycaprolactone (PCL) and Pluronic F127 [18,32].

#### 2.1.6. Rheological Properties

**Figure 3:**
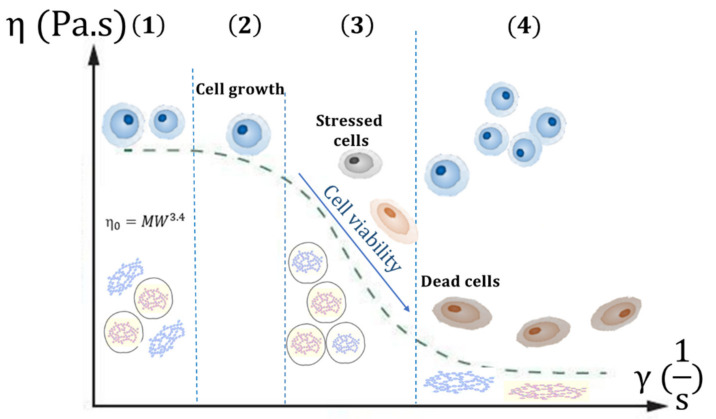
Shear−viscosity profile and respective cell behavior: $\left(1\right)\;\mathrm{Zero}\;\mathrm{shear}\;\mathrm{viscosity}$; (2); Polydispersity –increases with broader MW; (3) Shear-thinning—Linear region—ideal conditions for extrusion; (4) Infinite shear-rate [133,134,135].

While bioinks need enough viscosity to maintain its mechanical integrity when bioprinted, highly viscous formulations require higher extrusion pressures (resulting in higher shear stress). Although materials with G′ > G″ are more printable (with commercial available bioinks 0.30<δ < 0.45 [128]), a tradeoff between the two components of the dynamic modulus is required [133,134]. Moreover, bioinks acquire a shear-thinning behavior which consists of the slow reduction of viscosity when subjected to a deforming force and is characteristic of non-Newtonian fluids. On the other hand, this process must not compromise the cell viability if a cell-embedding strategy is used. Shear stress can influence cell behavior since moderate shear stress can influence cell differentiation while excessive stress damages cell function [135]. MW influences shear thinning functioning and subsequent cell viability and growth, and thus depends on the protein selected and extraction method used. (Figure 3).

In the herein reviewed studies on fibroin, elastin, fibrin, collagen and gelatin-based materials, the developed materials exhibited a shear-thinning behavior which is usually achieved by the addition of other components and crosslinking mechanisms [97]. In particular, gelatin has good rheological properties due to its reversible gelation, thus being often used with other proteins to improve the overall rheological behavior.

In the work of Trucco et al. [97] fibroin/alginate bioinks shear thinning properties were obtained between 1.0 and 1000 s^−1^. While the shear profile of the control fibroin-solution remained constant with shear rate (0.002 Pa·s), by adding gelatin crosslinked with tyrosinase, the viscosity was higher (1.71 Pa·s) and decreased with increasing shear rate. The same occurred in the works of Lee [62] and Freeman [109], where the addition of gelatin in GelMa/human-Recombinant-Elastin and fibrinogen hydrogels, respectively, gave the final formulation a shear thinning behavior. The non-Newtonian behavior of this bioink was also evidenced by its G′ over the loss modulus G″. Furthermore, when gelatin temperature increases, the viscosity decreases [62]. A different behavior was displayed in collagen bioink developed by Osidak and research team, in which the heating led to a slight decrease of G′ and G′ [136]. In the work of Mazzocchi et al. [128] methacrylated collagen and thiolated hyaluronic acid with good rheological properties were obtained (0.29 < δ < 0.33). These properties were further improved after UV mediated crosslinking, which resulted in higher G′ [128]. G” on the other hand did not suffer significant variations. Different methods to improve the rheological properties of collagen-based bioinks were proposed in different works and included TCPs addition [84], increasing the concentration of tannic acid [85]. Adding dECM to fibrin bioinks can also improve the final rheological properties and shear thinning behavior. Printability with 1% dECM-fibrinogen resulted in significant improvements in printability verified in crosshatch pore size, wall thickness and deflection from the overhang structure when compared with fibrinogen only and fibrinogen with 2% dECM [75]. It is clear that the rheological characteristics of the developed bioink formulation will determine the best printing settings [3,40,91].

### 2.2. Processing and Pos-Processing

#### 2.2.1. Printing Parameters

Once the bioink is formulated and characterized, it is essential to optimize the printing settings as a mean of maximizing resolution, shape fidelity, printability, reproducibility and to assure adequate cell behavior inside the hydrogel (Table 3).

The printing parameters are correlated with the printing method chosen with some being transversal to most techniques. Nozzle diameter and temperature, extrusion speed, bed temperature, and dispensation pressure are some of the parameters to consider and optimize based on the formulated bioink. For instance, increase dispensation pressure can be used when the bioink presents higher viscosity, which could be advantageous since more viscose inks typically can produce scaffolds with higher shape fidelity and allow more layering. However, the pressure can induce increase shear stress on the printed cells and induce cell apoptosis. In fact, the main challenges associated with extrusion bioprinting is the compromised cell viability under the shear stress-induced deformation during ink deposition. Induced shear rate, which is dependent of both rheological properties and printing parameters, determines shear stress applied to printed cells, influencing their viability [137]. As such, a balance between the several parameters that can be adjusted within the chosen printing technique and the bioink formulated must be met. Additional considerations should be placed on the limitations (e.g., extrusion pressure) inherent to each available equipment [37,42].

Extrusion bioprinting systems use a pneumatic system to dispense bioinks through nozzles that move according to a computer-generated toolpath. Extrusion pressure needs to be sufficient to overcome the surface tension of the bioink and in protein-based materials are usually <100 KPa [138]. While low extrusion pressures can lead to the formation of discontinuities in printed filaments, high pressures can cause flow instability. This parameter is highly dependent on yield stress and viscosity. In the work of Lee et al. [62], shear stress increased from 0.79 to 1.17 kPa, when the extrusion of methacryloyl-substituted recombinant human tropoelastin/GelMA pressure increased from 5 kPa to 25 kPa. The correlation between viscosity and extrusion pressure was reported by Yeo and colleagues [85]. Collagen-based bioinks with different rheological properties were achieved by varying the concentration of the crosslinker (1–3 wt% of tannic acid). Higher crosslinker concentrations were associated with higher storage modulus and required higher extrusion pressure, which is corroborated by the Hagen–Poiseuille equation [85]. The pressure used in systems which perform post printing cell addition is not as critical [3]. As extrusion pressure increases, the flow rate (amount of material extruded from the nozzle in volume/time) and feed rate (also referred to as printing speed) must increase as well. When the optimization process is initiated, a feed rate that allows the formation of a slow but a steady filament is desirable (~5 mm/s) [85,137]. In order to obtain a 3D structure, the bioprinter must allow displacement along the x-y plane, which can be achieved through the printhead (Table 3 (1)) or the working plane (Table 3 (2)). The speed of either of these components is linked to the overall bioprinting time, which should not be too long to avoid cell damage. Conventional reported values are ≤100 mm/s [84,139]. Higher values can also be adopted as in the work of Gao et al. [134], in which gelatin-alginate composite bioinks were fabricated with 150 mm/s using pressures from 60–70 KPa. Moreover, in protein materials, when fiber alignment is needed, the increase in flowrate can cause the protein to flow in the transverse direction at the nozzle tip, reducing the entropy, as described for collagen when reducing flow rate from 5 to 15 mm/s in the work of Kim et al. [131].

The increase in feed rate (1–6 mm/s) is reported to have a more significant effect in 3D alginate/gelatin constructs width and accuracy at higher extrusion pressure (250 KPa), leading to its exponential decrease [91]. The viscosity of the bionk also influences printing speed since higher viscosities require lower feed rates [91].

The flowrate is less described in the literature [91,131,137,139] However, this is an important parameter specially in protein-based bioinks due to its viscous properties. Bioinks with higher viscosity require a greater printing pressure to maintain the given printing flow rate [133]. Increasing the pressure can prompt high levels of shear stress during the printing process that can affect both immediate and long-term cell viability and proliferation [134].

Nozzle design such as diameter, length and shape also influence the filament characteristics. In the work of Trucco et al. [97] bioprinting of silk fibroin/alginate filaments with fixed pressure and speed (3 × 10^5^ Pa and 0.5 × 10^−3^ m/s) was used to investigate the effect of nozzles properties in the width of the final 3D construct. For the same diameter, the width decreased by increasing the length (6.35–25.4 mm), and for the same length, the width increased by increasing the diameter (0.05–1 mm) [97]. Moreover, in the study of Müller et al. [140] and Piard et al. [141] it was found that cell proliferation can be hindered by smaller nozzle diameter (<260 µm), probably to the increasing shear stress [140,142]. The distance between the nozzle and the bioprinting bed, also known as z-stepping height between layers, also conditions the properties of the final 3D construct. This parameter should be fixed accordingly to the nozzle diameter (distance ≈ ½ diameter nozzle) [143]. Regarding the geometry, Piard [141] further showed that at low pressures (<2 bar) conical needles result in higher cell viability, since higher shear stresses were only registered near fluid outlet. On the other hand, at high pressure (>2.5), cylindrical needles are preferable since as flow velocity increases the passage time of cells in this high shear region is reduced [141].

The temperature used during bioprinting is also a key factor for printing optimization, being used to modify the rheology of temperature-sensitive protein-driven bioinks (such as gelatin, collagen and protein/alginate hydrogels) to achieve greater control over printing. However, this setting is often overlooked and non-reported [87]. Both the cartridge temperature (internal temperature inside the printhead) and bed temperature (substrate temperature where the bioink is deposited) should be considered. Cartridge temperature is inversely related to hydrogel viscosity as temperature increase leads to lower viscosity, which induces less shear stress [3]. In protein bioinks the temperatures used are normally low when compared to synthetic materials, in order to mimic physiological conditions and to prevent protein denaturation. Bed temperature is usually similar to the one used in the printhead to avoid thermal shock. The most conventionally used temperatures in fibroin, collagen and gelatin-based bioinks are around 37 °C [85,131,136] and room temperature [104,106,107,144]. In our opinion, as room temperature oscillates (20–25 °C), defining a fix temperature is important to guarantee reproducibility. Temperatures close to 30 °C can also be adopted for collagen-based bioinks as the elasticity of collagen may decline significantly over the temperature range of 32–37 °C due to collapse of the triple-helix arrangement of collagen. In the work of Yeo et al. [85] near 35 °C the storage modulus of a collagen/polyphenol bioink significantly decreased [85]. Other approaches followed by Kim et al. [131] consisted on using 25 °C in the region of nozzle and 37 °C at the working plate.

Elastin and fibrin-based bioinks can adopt lower temperatures. In the work of Lee el at., [62] tropoelastin was printed using temperatures between 8 and 10 °C to achieve coacervation [62], while in the work of Wang et al. [32] the bioprinter was kept at 18 °C to produce bioinks with fibrinogen/gelatin/glycerol/hyaluronic acid.

It is also worth mentioning that the characteristics of the first bioprinted layer usually differ for the subsequent levels since continuous extrusion of bioink filaments is occurring and the base substrate ceases to be the bioprinter bed. Thus, a separated protocol should be developed for this first layer as proposed by O’Connell and co-authors [143].

#### 2.2.2. Adding Cells to the System

By definition a bioink is formed by combining cells with the established hydrogel formulation, to produce engineered live tissues using 3D printing technology [138]. Thus, cell incorporation is an essential processing step that not only contemplates the choice of cell type(s) and maturity but also cell density. However, there are several works in which the term “bioink” is used to describe a protein-based hydrogel with good biological performance demonstrated through in vitro assays [38,145]. In fact, conventional approaches involve cell embedding or subsequent cell seeding on 3D material after printing has also been reported [40]. A consensus on the right definition for the bioink concept is still needed.

**Figure 4:**
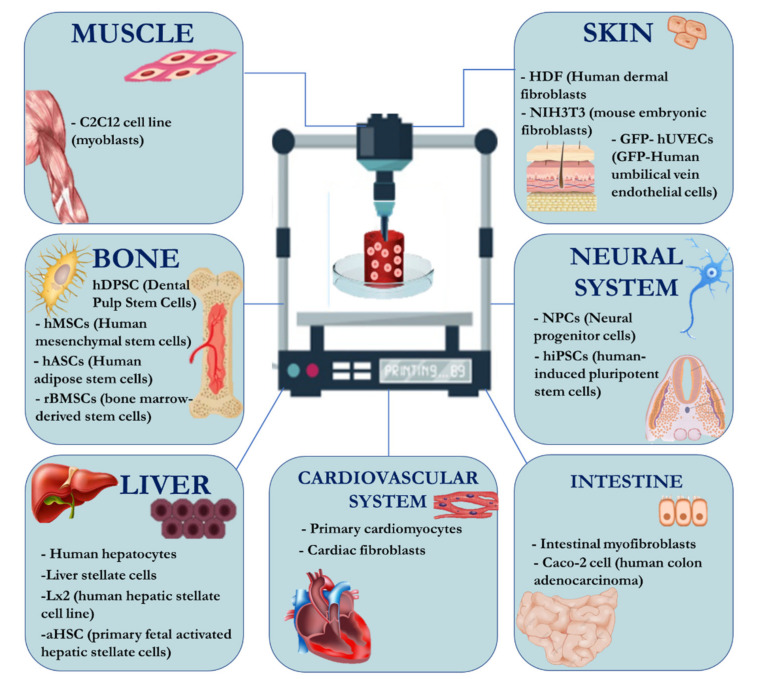
Conventional cell types selected for cell embedding in protein-based composites according and biomedical application.

Extrusion bioprinting is particularly suitable for bioinks with high cell densities (in the order of 10^5^–10^7^ cells/mL) and allows an excellent preservation of cell viability (40–80% post-printing viability is usually observed) (Figure 4) (Table 4) [104].

Cell embedding affects more than anything the rheology of the final hydrogel. As such, it should be evaluated after cell embedding. This occurs because cells within a bioink occupy a certain volume, which is dependent on cell type, size and selected density. The occupied volume is precluded to the hydrogel and may affect cross-linking efficiency and viscoelastic properties [147]. However, most studies lack on the evaluation of rheological properties after cell embedding. Piard et al. [141] correlated cell density with fiber density when using a 400 µm nozzle and showed that higher cell density resulted in viscous fibers with smaller diameter. However, when using a 250 µm nozzle, this effect was not evidenced. These findings suggest that viscosity alterations occur in function of the applied stress. Regarding shape retention over time, fibroin and gelatin-based materials were found to display excellent performance in the herein reviewed literature [97,106]. For example, in case of in silk-fibroin [106] and fibroin/gelatin bioinks [97] no changes were registered in construct dimensions or topography after nine weeks of bioprinting with intestinal myofibroblasts and after 28 days with human mesenchymal stromal cells (hMSCs), respectively. In this last case, hMSCs undewent osteogenic differentiation exhibited by the production of bone.

In elastin-based materials, incorporation with different cell types also exhibited excellent properties. Wu et al. [63] developed an elastin-like recombinamer bioink seeded with 10^5^ cells/mL of GFP-Human umbilical vein endothelial cells prevented cell viability (>90%) after 48 h of culture. As cell density decreases (10^3^), cell viability decreased proportionally (∼30%). Moreover, capillary-like structures and tube walls were obtained [63]. Complex structures with elastin were also reported by Duarte et al. [87]. To do so, the bioink with neural progenitor cells (NPCs) was directly dispensed onto endothelialized on-chip platforms. Prolonged cultures up to 2 weeks showed that NPCs spread continued growing (88.9%) and the endothelialized channels showed distribution of endothelial cells along the entire lumen of the channel. Contrastingly, survival of encapsulated human cancer spheroids (MCF10AT), after bioprinting was significantly lower compared to NPCs (70.2%), probably to the increase shear stress created by the increasing diameter (10 to 50 µm) [87]. NPCs were also used to incorporate with fibrin/alginate/chitosan bioinks. Cell viability increased from 90 to 97% in the first week and further in vitro studies allowed to identify neuronal/midbrain and dopamine/glial markers after 15 and 30 days, respectively [30].

Fibrin/alginate-based bioinks were effectively seeded with different cell types. By adding primary neonatal human dermal fibroblasts (HDF-n), collagen deposition and mechanical strength increased during two months of the cultures. The burst pressure of the vessel constructs reached 1110 mmHg, which is about 52% of the value of the human saphenous vein [109]. The addition of primary cardiomyocytes was also proven to be adequate to obtain complex constructs when combined with notch signaling blockade, an important factor in heart development and regeneration, which significantly accelerated development and maturation of uniformly aligned and electromechanically coupled cardiac cells [32]. In addition to alginate, the addition of other protein-driven components can also improve in vitro behavior. In the work of Jorgensen et al. [75], it was verified that by adding dECM to a fibrin-based bioink, the resulting constructs had greater structural stability and improved cellularity over time when compared with fibrin constructs.

Bone-related cells such as chondrocytes, MC3T3, bone marrow-derived stem cells (rBMSCs) were mainly added to collagen-based bioinks, as this protein is the main organic component of biological bone mineral [26]. Collagen/agarose effectively suppressed dedifferentiation of chondrocytes and preserved the phenotype [39,98] were able to maintained rBMSCs viability and proliferation up to seven days of culture [146]. When loaded with TCP, cell-viability 4 h after printing was >90% when particle amount was kept <20 wt%. When added in higher quantities, cells in the printing nozzle were severally damaged by the wall shear stress caused by the higher viscosity of the bioink [84].

Other innovative applications involving protein-based complexes have been reported. Kim et al. [76] combined decellularized small intestine submucosa (SIS) and collagen I to achieve a more realist physiological small intestine model and verified that adding dECM significant improved cell proliferation and generated a more meaningful epithelium layer mimicking the intestinal structure with capillary network formation when compared to pure cell-laden collagen. Moreover, meaningful results for various cellular activities, such as the expression of tight junction proteins, permeability coefficient and glucose uptake ability improved, compared with the pure 3D collagen villus structure [76].

Common problems associated with cell embedding include, as previously mentioned, cell damage due to shear stress, and cell sedimentation causing a non-uniform cell distribution within the bioink. The latter being special relevant in extrusion systems due to the characteristic high cell densities. In the work of Chen and authors [104], in order to circumvent these issues, a fibroin-GelMa bioink with high viscosity (liquid state) showed to provide interfacial retention, which retards the sedimentation of cells, but was also able to maintain a suitable biocompatibility [104]. Vascularization of 3D constructs is also an imminent concern which has been extensively addressed. Some studies use 3D bioprinting in combination with other technologies to increase the complexity of the developed tissue-like construct. In Duarte et al. [87] work, bioprinting was conducted in tissue-on-chip platforms to obtain endothelialized channels. A 3D intestinal villi micro vascular—vascular network was reported by Kim [103], using a dual-cell printing system supplemented with a core- shell nozzle. However, in simpler bioprinter systems, vascularization and tissue maturation can be achieved in post-processing. Bioreactors have shown to improve mass transfer of nutrients and gases in printer tissue constructs [17].

In vivo studies are still very scarce, which can be justified by the fact that many works intend to develop in vitro models. However, studies conducted over the last 3 years reveal promising results. Elastin-based bionks with over 85% cell viability after 7 days of culture do not cause inflammatory responses and have a good biodegradation rate when implanted subcutaneously in rats [62]. In this work, two printheads with different cells (cardiomyocytes (CMs) with cardiac fibroblasts (CFs) and human umbilical vein endothelial cells (HUVECs)) were used to obtain a final 3D material that demonstrated endothelium barrier function and spontaneous beating of cardiac muscle cells in vitro. Fibrinogen/gelatin bioinks with HUVECs and MSCs printed in osteon-like patterns and cultured in vitro in the work conducted in 2020 by Piard et al. [141] displayed significant increase in gene expression of angiogenic markers (BMP2, ALP, VEGFA and PECAM1) and cell viability >90% after 2 weeks. Histological tests performed after implanting, subcutaneously, the developws material in rats, showed a significant increase in the number of blood vessels per area [141]. Besides elastin, fibrin and gelatin, bioinks with collagen were also submitted to preliminary in vivo studies. Kim research team [131] loaded collagen with gold oriented nanowires and myoblasts (C2C12 cell line), and successfully upregulated the muscle tissue regeneration after in vivo implantation in rats [131]. The addition of dECM to silk-fibroin bioinks can further improve deposition of collagen and glycosaminoglycans. Zang et al. [74] showed that characteristic cartilage lacunas and round chondrocyte-like cells were evidenced 28 days after transplantation, contrary to what was observed in silk fibroin scaffolds. Additionally, expression of chondrogenesis-specific genes was found to be higher. Thus, fibroin/dECM scaffolds have promoted chondrogenesis and cartilage regeneration in vivo [74].

Biofabrication is also moving towards the possibility of in situ tissue regeneration. The in situ concept can be defined as the formation of a tissue-specific biomaterial (with or without cells and/or biomolecules) at the tissue defect where interactions will occur with the surrounding environment [148]. A recent example of an in situ printing extrusion system has been reported by Hakimi et al. [149] for skin tissue engineering. This hand-held bioprinter has enabled the in situ formation of biomaterials and skin tissue sheets of different architectures and compositions. The authors demonstrated that the device could allow for the bioprinting of single and multilayered biomaterial sheets with different biopolymers (alginate, fibrin, collagen, and hyaluronic acid) and cell types (fibroblasts and keratinocytes). The in vitro and in vivo experiments demonstrated that skin architecture was mimicked and that it is possible to cover large, wounded areas [149]. However, this strategy still faces many challenges, such as the need to scan the damaged area to print according to the defect size and shape, the difficulties in bioprinting, very irregular surfaces or the guaranty that the in situ bioprinting does not damage the surrounding tissues.

A critical parameter in the development and application of bioinks is that ensuring terminal sterilization of the precursor polymers and hydrogel materials is a fundamental requirement in a bioink design. Current standard techniques (heat, irradiation and chemical sterilization) often prove deleterious for the material properties, which brings to light to the importance of finding an adequate sterilization protocol to ensure minimal impact on the mechanical and chemical properties of the bioink and consequently on its printability and cell embedding results [18,133,150]. In the literature, there is still a great amount of works that do not disclose the sterilization strategy adopted in their studies which, in our view, represents an important gap to the field. For those reported, the most used techniques for hydrogel sterilization in the context of bioprinting have been sterile filtering [91], UV and gamma irradiation [151] and autoclaving [152]. There are also some works reporting the use of ethanol, however this is not a terminal sterilization method [153]. Many of the precursor polymers for bioink fabrication (as those reported here) are chemically active and thermosensitive and therefore can be modified or degraded during sterilization. Therefore, there is clearly room for improvement. Recently emerging techniques, such as supercritical carbon dioxide (scCO_2_) could become a viable option for terminal sterilization. scCO_2_ has a high potential to be adopted as a standard sterilization method, not only since it works at low temperature but also because of its minimally reactive nature and ability to diffuse into complex shapes [154,155]. Therefore, there is a great potential for sterilizing protein-based hydrogels as those described in the present review for 3D bioprinting, especially those incorporating growth factors or dECM. This technique is currently being accessed by FDA for its viability as a new standard sterilization modality [156].

**Figure 5:**
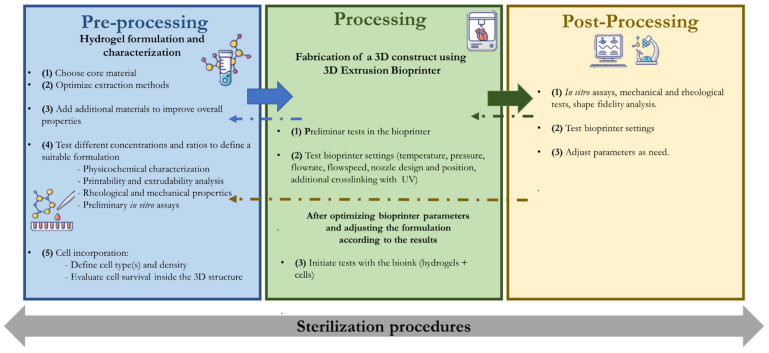
Schematic representation of the steps that must be considered in the development of a bioink.

Based on the parameters and research studies covered in this review, it is possible to identify a trend in the development and optimization of protein-based bioinks (Figure 5). The production of 3D constructs for biomedical applications using extrusion systems can be divided in (1) a pre-processing stage related to the design, formulation, and characterization of the materials to be printed; (2) a processing or printing stage, when the printing parameters are tested and put into use; (3) and finally a post-processing stage, where the printed structure is tested for its properties. Since every stage of this process has an impact on the properties of the printed structure, it is mandatory to go back to the previous stage and optimize parameters until the intended structure properties are reached. On the other hand, assuring sterilization is a concern which is transversal to all of the stages. The use of mathematical models to create the best experimental designs in 3D bioprinting has been also an important topic of research, to understand the relative importance of each parameter and to guarantee the best processing results possible [157,158].

## 3. Conclusions and Future Perspectives

Protein-based materials are emerging as one of the most promising sources for bioink formulations used in 3D printing technologies, being possible to fabricate biomaterials with a wide range of specific properties. The intrinsic mechanical strength and elasticity of natural proteins such as silk fibroin, elastin, resilin, collagen, and gelatin have put these proteins at the center of such research. Nevertheless, one of the biggest challenges in using proteins is their batch-to-batch variability. Therefore, it is important to establish strict and well-defined protocols for the extraction, concentration, and purification of these proteins in order to ensure that results are reproducible and reliable.

Current proteins sourced from xenogeneic sources should be weighed against its allogenic counterparts. Xenogeneic sources present ethical concerns and increase risk of pathogen transmission, additionally regulatory bodies have strengthened the importance of moving away from animal methods (e.g., Regulation No 1907/2006, EU Directive No 2010/63/EU). However, allograph sources could present increased difficulty in availability of source material specially when considering regulations on organ and tissue donation specific to each country. If moving towards allogenic sourcing, companies, research groups and regulatory bodies should work together towards achieving more standard regulations on organ and tissue donation. The proteins here discussed in detail, namely collagen, gelatin, fibroin, fibrin, elastin and dECM are those that have reached the furthest in the bioink development pipeline. However, other proteins such as resilin, silk sericin, keratin, have already been successfully used in the form of hydrogels for biomedical applications with promising in vitro results and in some cases effective 3D bioprinting. Thus, the tunning of the hydrogel to allow cell embedding and printability are crucial to take these hydrogel systems to the next level. Here, the choice of the best available sterilization method will play an important role for ensuring the best properties and safety of the final bioink and 3D construct.

Another aspect to consider is the printing technique to be explored. Despite the clear advantages associated with extrusion-based printing, this technique lacks in resolution. Since cell activity is influenced by topographical cues, exploring techniques with greater resolution, such as fused-deposition modeling [11,18,21], and stereolithography [6], that allow increased control over this feature should be addressed [42]. Another alternative for obtaining more complex structures and with important cues for cellular interaction and vascularization of the micro-tissue formed is the combination of extrusion bioprinting with other emerging technologies such as microfluidic to simulate real tissue functions, where bioinks can be printed with different types of cells [87]. More complex cell culture systems can provide mechanical and/or electrical stimulus expected in the human body in order to provide a maturation environment that closely resembles it.

The next generation of tissue engineered materials is expected to mimic not only the organs’ or tissues’ architecture and properties, but also their dynamic function [159,160]. Time is considered the fourth dimension (4D). This concept emerged associated with biofabrication and consequently with bioprinting, at two levels: materials capable of deformation and structures that mature after printing [160,161]. This new 4D bioprinting perspective reveals the complexity of the system which is essential to fully understand the behavior at the postprocessing stage of the processed functional living materials.

Successful examples of protein-based bioinks for the development of tissue engineered constructs using 3D biofabrication are herein described. While in vitro models have been reaching applications towards the study of diseases and the development of new therapies, the translation of this concept to the surgery room is not yet fully available. It is unquestionable however that great progress is currently being done in hydrogel design and on the development of advanced technological tools to reach the fidelity and safety requirements for bioprinted constructs to reach patients in a consistent and personalized manner in the near future.

## Figures and Tables

**Table 2:** Preprocessing parameters to consider throughout the development of bioink formulations and examples of possible generic characterization [3,18,40].

**Protein characteristics**	MW determination Protein sequencing Standardization of extraction protocols	Chromatography Mass spectrometry
**Preliminary in vitro assays**	Viability tests	Calcein AM Green Live Dead assay Propidum Iodide Alamar blue Hoechst 33342
	Metabolic tests	Cell Counting Kit-8 assay (CCK8) 3-[4,5-dimethylthiazol-2-yl]-2,5 diphenyl tetrazolium bromide assay (MTT) Positron emisson tomography activity (PET)
**Mechanical**	Compressive tests Young or elastic modulus Compression modulus Yield stress Ultimate tensile strength (UTS)	
**Quantitative measures (Extrudability)**	Viscosity Rheology Extrusion uniformity Structural integrity.	

**Table 3:** Printing conditions for protein-based bioinks extrusion.

Extrusion Bioprinter	Parameters	Units	Most Used
** 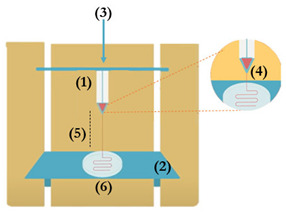 **	(1) Cartridge/Printhead temperature	°C	37 °C Room temperature
	(2) Bed/working plane temperature.		20 °C–40 °C
	(3) Extrusion pressure (pneumatic, piston, screw)	Pa (bar, psi, N/mm^2^)	4–150 KPa
	(4) Nozzle	µm	0.26 mm–250 mm
	Printing speed (Feed rate)	mm/s	0.2–150 mm/s (most common 1–30 mm/s)
	Flow rate	mm^3^/s	Not described
	(5) Distance between the nozzle and substrate	cm	½ nozzle diameter
	(6) 3D structure		- Conventional shapes: squares, rectangles and spheres - Fiber resolution in the microscale (µm)

**Table 4:** Overview of the application of cell embedding in 3D bio-printed materials.

Formulation	Cell Type	Cell Density (Cells/mL)	Cell Viability & Functionality	Ref.
Gelatin (5% *w*/*v*) and silk fibroin solution (5% *w*/*v*)	hMSCs	1 × 10^7^	After 28 days: 95% cell viability and increase of SOX-9, the specific chondrogenic transcriptional factor, as well as of collagen type 2.	[97]
MeTro (5 % *w*/*v*), GelMA (7.5% *w*/*v*) and gelatin (20% *w*/*v*)	CMs, CFs, HUVECs	2 × 10^7^	After 7 days of culture: 85% cell viability. Endothelium barrier function and spontaneous beating of cardiac muscle cells.	[62]
Elastin-like protein (3, 4, or 5 % *w*/*v*)	NPCs	1 × 10^7^	After 7 days of culture: 88.3% cell viability. - after 5 days of presence of hiPSC-NPCs (Sox-2-positive, a marker of neural progenitor cell pluripotency)	[87]
Elastin recombinamer (2 % *w*/*v*) + graphene oxide (GO) (0.1 % *w*/*v*)	GFP- hUVECs	5 × 10^3^, 1 × 10^4^, 5 × 10^4^, and 1 × 10^5^	After 48 h of culture, membranes exhibited different cell viabilities ∼30%, 50%, 80%, and >95% (according to the different cell densities used)	[63]
Gelatin (7.5 % *w*/*v*) and 10 mg/mL fibrinogen	HDF-n	3 × 10^6^	80% cell viability after printing	[109]
Fibrinogen (20 mg/mL), gelatin (30 mg/mL), aprotinin (20 μg/mL), glycerol (10% 1/v), and hyaluronic acid (3 mg/mL)	Primary cardiomyocyte	10 × 10^6^	Progressive cardiac tissue development was confirmed by immunostaining for α-actinin and connexin 43	[32]
Fibrinogen (20 mg/mL), 0.5 (% *w*/*v*) of alginate and genipin (0.3 mg/mL)	NPCs	1 × 10^6^	After 7 days of printing: 95%. - The bioprinted tissues expressed the early neuronal marker, TUJ1 and the early midbrain marker, Forkhead Box A2 (FOXA2) after 15 days of culture. Other glial markers such as glial fibrillary acidic protein and oligodendrocyte progenitor marker were present after 30 days.	[30]
Fibrin (20 mg/ mL), alginate (5 mg/mL), genipin (0.3 mg/mL).	hiPSCs	1 × 10^4^	After 10 days: 94.72% cell viability. After 15 days: 64.12%. - hiPSCs in the presence of puro and RA differentiated into neurons as indicated by early neuronal expression marker TUJ1 and the long neurite extensions into the scaffold.	[111]
Fibrinogen (10% *w*/*v*) and (5% *w*/*v*)	hMSCs and HUVECs	2 × 10^6^	After 7 days of culture: 94.8% cell viability. - Increase in gene expression of BMP2, ALP, VEGFA and PECAM1.	[141]
Fibrinogen (5–20 mg/mL), gelatin (37.5 mg/mL), hyaluronic acid (3 mg/mL), and glycerol (4% *v*/*v*)	hDPSC	-	After 7 days of culture: >90% cell viability	[31]
Collagen (5 *w*/*v*%) and mixed Au nanowires (GNWs)	myoblasts (C2C12 cell line)	1 × 10^7^	After 1 days of culture: >90%.	[131]
Collagen (15 mg/mL), agarose (1:4) mixed with sodium alginate (0.1 g/mL)	chondrocytes	1 × 10^7^	- High cell viability after culture for 14 days (>80%). - Expression of cartilage specific genes such as Acan, Col2al and Sox9.	[98]
Collagen branded Viscoll (2, 3 and 4 *w*/*v*)	NIH 3T3 fibroblasts	0.5 × 10^6^	After 7 days of culture: 97.2%, 95.2% and 87.2% for bioinks at 2, 3 and 4% collagen concentrations	[136]
Collagen (5 % *w*/*v*) mixed with various weight fractions of β-TCP (0, 10, 20, and 45% *w*/*v*)	MC3T3-E1	1 × 10^7^	After 7 days of culture: 92% cell viability. For greater than 20 wt% of β-TCP, the cell-viability was significantly lowered.	[84]
Collagen (4 % *w*/*v*)	hASCs	2 × 10^6^	After 5 days of culture: 93% cell viability.	[85]
Collagen (5 mg/mL) and tyramine derivative of hyaluronan (25 mg/mL)	hMSC	3 × 10^6^	- The cells were viable after printing and remained viable over culture time. After 6 days, the number of dead cells were less than that observed on day 1. - hMSCs embedded in the isotropic bioink displayed chondrogenic differentiation comparable	[107]
Collagen (3 mg/mL)	rBMSCs	10 × 10^6^	Cell viability recorded were >88% after printing.	[146]
Fibrinogen (30 mg/mL), gelatin (35 mg/mL), glycerol (100 μL/mL), and hyaluronic acid (3 mg/mL)	Human keratinocytes, melanocytes, fibroblasts, dermal microvascular endothelial cells, follicle dermal papilla cells, and adipocytes	20 × 10^6^	All bioprinted skin treated wounds closed by day 21, compared with open control wounds. Wound	[110]
Collagen solution (4, 8, or 12 mg/mL) and riboflavin	chondrocytes	1 × 10^6^	After 7 days of culture: >95% cell viability at all collagen concentrations tested. However, the addition of blue light activated riboflavin crosslinking decreased viability to 76–77%.	[39]
Collagen-based (4 % *w*/*v*) and tannic acid (0, 1, and 2 % *w*/*v*)	Caco-2	5 × 10^6^	After 7 days of culture: 93% cell viability.	[103]
